# External Mill Monitoring of Wheat Flour Fortification Programs: An Approach for Program Managers Using Experiences from Jordan

**DOI:** 10.3390/nu5114741

**Published:** 2013-11-21

**Authors:** James P. Wirth, Erin Nichols, Hanan Mas’d, Rawhieh Barham, Quentin W. Johnson, Mary Serdula

**Affiliations:** 1 GroundWork LLC, 40b Les Landes, Crans-près-Céligny 1299, Switzerland; 2 Global Alliance for Improved Nutrition, Geneva 1211, Switzerland; 3 Epidemic Intelligence Service assigned to the Division of Nutrition, Physical Activity, and Obesity, Centers for Disease Control and Prevention, United States Public Health Service, Atlanta, GA 30333, USA; E-Mail: igd1@cdc.gov; 4 Nutrition Division, Department of Non-Communicable Disease, Ministry of Health, Amman 11118, Jordan; E-Mails: Hanan-MoH@live.com (H.M.); majeda_barham@hotmail.com (R.B.); 5 Training and Technical Support Group, Flour Fortification Initiative, Atlanta, GA 30322, USA; E-Mail: quentin@quican.com; 6 Division of Nutrition, Physical Activity, and Obesity, Centers for Disease Control and Prevention, Atlanta, GA 30333, USA; E-Mail: mks1@cdc.gov

**Keywords:** fortification, external monitoring, wheat flour, compliance, micronutrients

## Abstract

The fortification of wheat flour with micronutrients is a common strategy to increase vitamin and mineral intake. While wheat flour mills are often inspected by agencies affiliated with national ministries to ensure compliance with national fortification standards, few countries use data derived from these inspections to construct an external monitoring system for use in program management and evaluation. The primary objective of this paper is to assess the performance of the external monitoring system utilized in Jordan according to the US Centers for Disease Control and Prevention’s Updated Guidelines for Evaluating Public Health Surveillance Systems. A secondary objective is to present mill monitoring results from 2009 to 2010 in order to demonstrate the data generated by the system. The review concludes that the data required for the system is representative, simple to collect, and can be collected in a flexible manner. The external monitoring system is acceptable to participating agencies and millers and is stable due to mandatory fortification legislation which provides the legal framework for external monitoring. Data on production of fortified flour and utilization of premix can be provided in a timely manner, but on-site mill monitoring and flour sample collection are more challenging due to resource constraints. The frequent collection of a small number of indicators can provide fortification program managers with timely information with which to base decisions. Jordan’s external monitoring system successfully documented the performance of each mill and the entire flour fortification program, and can serve as a model for other national fortification programs considering external monitoring approaches.

## 1. Introduction

The fortification of wheat flour with micronutrients is a common strategy to increase vitamin and mineral intake and is included as a key component in national health and nutrition strategies [1]. As part of a national fortification program, a country’s milling industry is often required to add micronutrient premix in defined doses to the flour (typically wheat or maize) they produce. While many national fortification programs require that a set premix formulation be utilized, some programs only specifically require that the quantity of nutrient compounds that must be present in the final product (flour). Under this approach, each mill can procure different micronutrient premix blend and add them in appropriate amounts to meet national standards. In order to ensure that the dosing of the micronutrient premix is properly conducted, internal (*i.e*., conducted by millers) quality assurance (QA) processes and quality control (QC) tests are employed during production. QA/QC processes and tests related to fortification verify that fortification is properly implemented and identify any irregularities in the dosing and mixture of the micronutrient premix [2]. In addition to internal measures to assure quality, external monitoring measures are often implemented by national or sub-national agencies affiliated with national ministries of health to ensure that mills comply with national fortification standards [3]. This is of particular concern where the fortification of flour is mandatory [4] or the government plays an active role in the implementation of the fortification program.

With respect to flour fortification, the term external monitoring is used to describe factory inspection and technical auditing by the government food control unit [1]. External monitoring can also include conformity assessments whereby flour samples are periodically collected by a food control unit to determine if they adhere to fortification specifications [1]. The data collected can be used in one of two ways: (1) to pass or fail individual mills based on the data collected from them, and/or to (2) establish an external monitoring (surveillance) system whereby the data from all mills are routinely collected and analyzed in order to be used for implementing and evaluating the fortification program.

Literature detailing the processes associated with external monitoring exists but is limited. Peña-Rosas *et al*. [3] developed a practical approach for designing a monitoring and evaluation system for flour fortification programs and review both mill-level and external monitoring processes, and the World Health Organization (WHO) describes the rationale and theory underpinning external monitoring of food fortification programs [1]. External monitoring systems are also discussed in various manuals pertaining to food fortification [5,6,7,8,9], though these manuals are primarily focused on QA/QC processes and tests conducted at the mill-level. Managers of national fortification programs must be able to identify and respond to poor compliance by millers and are required to make critical decisions related to fortification programs, such as budgeting for the purchase of key program inputs (e.g., micronutrient premix feeders and premix) and the implementation of surveys and impact evaluations [1]. As few managerial approaches to monitoring food fortification programs have been detailed in the peer-reviewed literature, there is a clear need to assess the performance of on-going external monitoring systems in order that “lessons learned” be documented and communicated more broadly. The US Centers for Disease Control and Prevention’s (CDC) have developed general criteria for evaluating monitoring and surveillance systems in order to promote the best use of resources by ensuring that monitoring systems operate efficiently and serve a useful function that meets the system’s objectives.

This paper assesses the quality of Jordan’s external monitoring system and evaluates the performance of the national wheat fortification program using data collected by this system. Jordan has one of the most comprehensive wheat flour premix formulations utilized today, and based on the WHO 2009 statement on wheat and maize flour fortification, Jordan’s premix formulation contains the recommended quantities of iron, folic acid, and vitamin A for populations where the average flour consumption is 150–300 g per day [10]. Moreover, a 2010 review of the iron (type, quantity, and relation to diet) utilized by national fortification programs noted that Jordan was one of the few countries where a “significant positive impact on iron status” would be expected [4]. As such, the impact of the fortification program relies heavily on the proper implementation of the program and successful compliance to national standards. Furthermore, as there are few published descriptions of external monitoring systems linked to flour fortification programs, a review of Jordan’s monitoring system offers a unique perspective of how external monitoring data are used to track program progress and inform decision makers.

## 2. Objectives

The objectives of this paper are (1) to assess the external monitoring approach utilized by the Jordanian government to monitor the national wheat fortification program against the CDC guidelines of evaluating surveillance systems [11] and (2) to present the mill monitoring results from 2009 to 2010 in order to demonstrate the data generated by the system. It is hoped that a thorough exploration of this approach will prove useful to managers of wheat flour fortification programs in other countries.

## 3. Background of Fortification in Jordan

### 3.1. Wheat Flour and Micronutrient Premix

The type of flour being fortified in Jordan is *Mowahad* wheat flour. *Mowahad* wheat flour has an extraction rate of 73%–78% [12] and is the most widely consumed wheat flour in Jordan, with more than 90% of all wheat flour being *Mowahad* [13]. As one of the primary staples of the Jordanian diet, *Mowahad* wheat flour is the only flour subsidized by the government [13]. In 2002, the Jordanian Government passed legislation mandating that all *Mowahad* wheat flour be fortified according to national standards [13,14].

The flour fortification program in Jordan began in 2002 [15] with iron (dried ferrous sulfate) and folic acid included in the micronutrient premix. This micronutrient premix formulation was utilized until 2006 when the number of nutrients was increased to include zinc, and Vitamins A, B1, B2, B3, B6, and B12 [12]. The increase in the number of nutrients in 2006 was undertaken with the support of a small grant provided by the Global Alliance for Improved Nutrition. In 2010, Vitamin D was also added to the micronutrient premix formulation following studies showing considerable Vitamin D deficiency in Jordan [16] and neighboring countries [17]. Table 1 details the inclusion of additional nutrients to Jordan’s micronutrient premix from 2002 to 2011.

**Table 1:** Micronutrient premix standards in 2002, 2006, and 2011.

Nutrient (Compound)	Amount (ppm) in 2002	Amount (ppm) in 2006	Amount (ppm) in 2011
Iron (Ferrous sulfate)	30.00	32.25	32.25
Zinc (Zinc oxide)	n/a ^1^	20.00	20.00
B1 (Thiamin mononitrate)	n/a	3.575	3.575
B2 (Riboflavin)	n/a	3.60	3.60
B3 (Niacinamide)	n/a	35.00	35.00
B6 (Pyridoxine)	n/a	4.40	4.40
B9 (Folic acid)	1.50	1.50	1.50
B12 (Vitamin B12 0.1% WS ^2^)	n/a	0.007	0.007
Vitamin A (Vitamin A palmitate, SD ^3^)	n/a	1.50	1.50
Vitamin D (D3 Cholecalcerferol)	n/a	n/a	0.0145

^1^ n/a = Not Applicable, as specific micronutrient was not mandated at this time; ^2^ WS = Water Soluble; ^3^ SD = Spray Dried.

Since 2002, the government of Jordan has purchased equipment and has begun distributing micronutrient premix at no cost to all wheat flour mills in Jordan. The total annual cost to the government for the procurement of premix distributed to millers is approximately 1.2 million Jordanian Dinar (JD) [12] (~1.7 million USD), or approximately 0.19 JD (~0.27 USD) per capita.

The national wheat flour fortification program is managed by the Nutrition Division within the Jordanian Ministry of Health (MoH). The Nutrition Division, along with other relevant government agencies, is responsible for setting the fortification standards, the purchase and distribution of the micronutrient premix, and the monitoring of the national food fortification program.

### 3.2. Monitoring of the Fortification Program

The Jordanian government’s fortification monitoring system is used both to assess the performance of the fortification program and to make key programmatic decisions, such as the quantity of premix to purchase for the following year. From 2006 to 2008, data on the fortification program was compiled and analyzed on an annual basis. These annual reports included information related to the production of *Mowahad* wheat flour, the utilization of premix, and the quantitative testing of iron in bread. These reports were compiled retrospectively and primarily to estimate the budget required for the following year’s premix purchases. As these reports were comprised of retrospective data, they could not be used to address issues of non-compliance at the mill level in real time.

Though the monitoring system is coordinated by the MoH’s Nutrition Division, other agencies play an active role in the external monitoring processes through the food fortification Technical Committee. Specifically, the Technical Committee is comprised of individuals from the Ministry of Industry and Trade (MIT), Jordanian Food and Drugs Administration (JFDA), provincial health inspectors, and the MoH Nutrition Division, and is responsible for preparing and reviewing food safety and fortification regulations and for conducting mill inspections.

In 2009, a prospective monthly monitoring system was established to document the quality of the fortification program. This monitoring system aims to ensure the proper implementation of the flour fortification program. Though this system collects similar data and indicators as the reports generated between 2006 and 2008, its more frequent collection and analysis of the data now enables fortification program managers to address issues of compliance in real time.

The system consists of five indicators derived from three key data sources from which the adequacy of the fortification of each mill is assessed. Table 2 details the five indicators that comprise the mill monitoring system, the data sources underpinning each indicator, the indicator’s use, and the process utilized by the various agencies to submit/transfer to the data.

The quantity (metric tons) of “fortified” *Mowahad* wheat flour produced in the past month is sourced from mill production records (Indicator 1). In order to receive subsidies related to *Mowahad* wheat flour, mills are required to report the quantity of fortified flour produced to the MIT. As such, mills submit production reports, consisting of aggregated daily production data to the MIT via fax on a monthly basis.

The approximate utilization (number of 25 kg boxes) of micronutrient premix used in the past month is sourced from premix storage logs (Indicator 2). In order to ensure that mills will have sufficient micronutrient premix with which to fortify their wheat flour, the mills provide premix utilization to the MoH via fax or e-mail. With this information, the MoH can transport additional premix supplies to each mill when required, and plan for future premix orders.

**Table 2:** Monthly food fortification monitoring report.

Grouping	Indicator Name	Data Sources	Indicator Use	Data Transfer Process
Raw Information Collected	1. Monthly Production of *Mowahad* Wheat Flour	Mill production records	Used to calculate Average Addition Rate	Received by the MIT (via monthly fax or email) from each mill and sent to the MoH (via fax) from MIT upon request.
	2. Number of 25 kg Premix Boxes Utilized	Premix storage logs	Used to calculate Average Addition Rate	Millers, sent via monthly fax to MoH
Calculated indicators	3. Average Addition Rate	Calculated using mill production records and premix storage logs (see equation in Table 3)	Used to assess if the appropriate quantities of premix are being utilized.	n/a
	4. Addition Rate as a % of 250 g/MT target	Calculated using Average Addition Rate (see equation in Table 3)	Calculated to show the % of the target being achieved at the mill level by month and on average and, in aggregate, the national level.	n/a
External test of iron concentration	5. Iron concentration in flour sample	Randomly collected wheat flour sample	Used to determine the compliance to fortification standards at mill level, and to verify against calculated premix utilization results.	Flour samples collected by members of the Technical Committee or local MoH inspectors during monthly visits to mills and bakeries. Analysis of the flour samples is conducted by the JFDA, RSS, and FSLD laboratories.

**Table 3:** Example of monthly food fortification monitoring report. (February 1–28, 2010).

Mill Name (List ALL Mills Producing *Mowahad* Flour, Even if the Flour Is Not Currently Being Fortified)	*Mowahad* Flour Production (MT)	Premix Boxes (25 kg each)	Average Addition rate g/MT ^1^	Addition Rate % of Target 250 g ^2^	Iron Level PPM	Comments Describe Any Issues at Mills that Need to Be Addressed (e.g., Broken/Needed Parts, Testing Supplies Needed, *etc.*)
(A)	(B)	(C)	(D)	(E)	(F)	(G)
Mill 1	1962	18	229	91.6	60.3	
Mill 2	5116	30	147	58.6	32.0	
Mill 3	1970	18	228	91.4	45.26	
Mill 4	3000	27	225	90.0	40.9	
Mill 5	4694	41	218	87.0	35.5	
Mill 6	2576	10	97	38.8	12.7	
Mill 7	3496	32	229	91.5	21.9	
Mill 8	5982	28	117	46.8	21.2	
Mill 9	5735	45	196	78.5	24.9	
Mill 10	5543	42	189	75.6	28.2	
Mill 11	800	8	250	100.0	28.0	
Mill 12	2544	20	197	78.6	24.5	
Mill 13	-	-	-	-	-	No feeder in the mill

^1^ (D) = ((C) × 25 kg × 1000)/(B); ^2^ (E) = (D)/250.

From Indicators 1 and 2, the average addition rate (also known as incorporation rate; calculated as the number of grams of premix added to one MT of flour) for the premix is calculated to determine if the mills were using the appropriate amount of premix (Indicator 3). The target for the addition rate of the micronutrient premix is 250 g per metric ton of flour. This standardized addition rate assures that appropriate concentrations of micronutrients will be added to the flour. The average addition rate establishes a mill’s average usage of premix. Indicator 4 presents the average addition rate as a percentage of the 250 g/MT target.

The iron concentration of the mill’s flour is sourced from a quantitative test conducted on a composite wheat flour sample (Indicator 5). For this test, members of the Technical Committee of the food fortification program and locally-based MoH health inspectors collect between three and six flour samples from the end of a mill’s production line and from packed flour sacks as part of their routine monthly inspections. Flour samples from bakeries were also collected at times, though not systematically (e.g., every month). The Technical Committee members and locally-based MoH health inspectors coordinate their inspections based upon their proximity to a given mill. Individual samples are between 200 and 300 g in weight, with the resulting composite sample weighing ~1 kg. As part of these inspections, staffers also confirmed that the micronutrient premix is correctly stored and that the micronutrient premix feeders were functioning properly. Flour samples are analyzed at the JFDA, the Royal Scientific Society (RSS), and Forensic Science Laboratory Department (FSLD) using atomic absorption to assess the iron concentration.

The information for each mill is received by the MoH via fax or email and compiled into a standardized form. Table 3 presents the one-page example of a monthly monitoring report completed by staff members in the Jordan MoH’s Nutrition Division which shows production, premix utilization, addition rate, and iron content results for all of Jordan’s 13 mills.

## 4. Methods

The CDC’s Updated Guidelines for Evaluating Public Health Surveillance Systems [11] provides a framework for assessing the quality of public health monitoring systems and are utilized here to assess the performance of Jordan’s external mill monitoring system. According to these guidelines, various “system attributes” of the monitoring system should be examined qualitatively in order to ascertain the overall usefulness of the monitoring system.

The guidelines present nine general “attributes” of a public health monitoring system; namely simplicity, flexibility, data quality, acceptability, sensitivity, predictive value positive, representativeness, timeliness, and stability. According to the CDC guidelines, as monitoring systems “vary in methods, scope, purpose, objectives, *etc.*, public health surveillance system(s) should emphasize those attributes that are most important for the objectives of the system” [11]. With this understanding, the attributes “sensitivity” and “predictive value positive” were not selected for this review as they relate most directly to population-based data collection of the detection of disease. As such, seven system attributes (simplicity, flexibility, data quality, acceptability, representativeness, timeliness, and stability) were deemed relevant to the objectives of Jordan’s national fortification program and its external monitoring system and are discussed in detail in the results section. Based on this critique of the attributes, the overall usefulness of the monitoring system is presented in the discussion. The review of the system’s attributes and usefulness is based on the authors’ judgment and familiarity with the monitoring system’s operations and performance. In addition to qualitative data, quantitative mill monitoring results from 2009 to 2010 are also presented in order to demonstrate the data generated by the Jordan’s external monitoring system.

## 5. Results

### 5.1. Review of Monitoring System’s Attributes

Using the CDC framework for evaluation of surveillance systems [11], various attributes of the Jordanian fortification program’s mill monitoring system are presented in detail. As the CDC framework was established to evaluate the surveillance systems of various public health programs, only attributes relevant to food fortification programs are discussed here. These attributes include the monitoring system’s simplicity, flexibility, data quality, acceptability, representativeness, timeliness, and stability. For each system attribute, the data collection processes are described and critiqued.

#### 5.1.1. Simplicity

The external monitoring system involves the assessment of only a few simple indicators. Collecting the data required to track these indicators on a regular basis does not require complex methods or tools. The MIT receives data on the quantity (metric tons) of *Mowahad* wheat flour produced by each mill on a monthly basis by fax or e-mail. This information is submitted as part of the national subsidy scheme, and this information is shared with the MoH upon request. Data on premix utilization, sent via fax directly from the mills to the MoH, has been designed to be simple to produce. Rather than request the use of premix in kilograms, the system only requests the number of boxes of premix used in order to approximate the amount of premix used. As the quantitative testing of iron in the flour samples requires the collection of flour samples from each mill, the process is at times difficult to implement. The difficulty in collecting flour samples is due to the time required by members of the Technical Committee to travel to each mill, particularly mills that are far from Amman (where the members of the Technical Committee reside).

#### 5.1.2. Flexibility

With respect to the indicators collected through the monitoring system, there is only marginal flexibility, as the system focuses on the repeated collection of a few pre-established data points and is designed to be operated with minimal financial and technical requirements. Though additional indicators could be added into the monitoring system, this could not be done without increasing the complexity (*i.e.*, sacrificing simplicity) of the entire system.

Though indicators cannot be easily added to the system, the data collection process is quite flexible. For example, though the MoH can receive all mill results from the MIT, certain millers have also submitted the production results directly to MoH. Moreover, as the data points for each mill are few, these can be communicated using a variety of different methods, such as fax and e-mail. Lastly, there is flexibility in the frequency in which data can be collected, particularly once the mills within the system demonstrate regular compliance [1]. For example, though the Jordan monitoring system cangenerate monthly results, program managers can collect data less frequently once compliance is verified. In contrast, prior to program-wide activities, such as national surveys, or in the event that a mill is non compliant, the frequency of data collection can be returned to a monthly schedule.

#### 5.1.3. Data Quality

The quality of the data collected on quantity (metric tons) of *Mowahad* wheat flour is verified by MIT officials. As the subsidies to millers are linked to the provision of subsidized wheat grain, the MIT is able to verify if the reported production given by each mill is feasible. The quantity of premix utilized reported by the millers is verified by the MoH, as they purchase premix for the entire country and distribute to each mill. The quality related to quantitative testing of iron is high as composite samples are collected according to international recommendations [9] and are measured by laboratories that adhere to strict quality assurance procedures. The simplicity of the system and verification of external monitoring data (*i.e.*, calculated premix added and spectrophotometry results) contribute to good data quality. Data quality is currently limited by a lack of a database in which to compile data. A database would provide a means for automating the data review processes by simplifying the generation of key charts and tables to aid in the interpretation of data and by facilitating the tracking/summarizing of the trends over time.

#### 5.1.4. Acceptability

According to the CDC evaluation guidelines [11], acceptability is described as “the willingness of persons and organizations to participate in the surveillance system”. In this respect, program staff in the MoH Nutrition Division have confirmed [18] that the external monitoring system is acceptable due to its simplicity and dedicated financial support from the Jordanian MOH. However, the follow-up process for non-compliant mills demands additional resources in time and money. With respect to millers, the system is acceptable as their provision of regular data to the MoH and MIT facilitates their receipt of grain subsidies and free micronutrient premix.

#### 5.1.5. Representativeness

The monitoring system collates information from all mills that produce *Mowahad* wheat flour in Jordan and is therefore fully representative.

#### 5.1.6. Timeliness

The system is designed to collect data on a monthly basis, and between 2009 and 2010, data collection activities were conducted at acceptable levels. It is recommended that data on the production of fortified flour and utilization of premix should be conducted monthly [6], as this data enables program managers to address instances of non-compliance or technical problems faced by millers. Monthly on-site mill monitoring and flour sample collection, however, is a costly and time-consuming exercise. As the 2009–2010 mill monitoring results show (see Table 4), gaps in the collection of monthly monitoring data did occur. Compared to the monitoring undertaken between 2006 and 2008 during which data was analyzed retrospectively, the present monitoring system provides a better means of addressing issues in a timely manner.

#### 5.1.7. Stability

One key factor which provided stability to the system is the presence of legislation mandating flour fortification [4]. Such legislation provides the legal framework for external monitoring and compliance systems. In addition, dedicated funds from the Jordanian government to subsidize (via purchase of premix) and oversee the national wheat flour fortification program, add further stability to the system. Despite these dedicated funds, however, the fact that Jordan’s MoH employs only a small number of staffers to oversee all nutritional programs, thus limiting the attention that can be given to monitoring fortification, compromises the system’s stability.

### 5.2. 2009–2010 Mill Monitoring Results

Table 4 presents the consolidated results from the monthly mill monitoring reports from January 2009 to April 2010. Table 4 presents the monthly performance of each mill when premix stocks were available, and where there were gaps in the implementation of the fortification program or when information was not collected. For example, the micronutrient premix stock was not present in the mills in February, May, and between July and October 2009. The gap in the provision of micronutrient premix occurred because the premix delivered in July 2009 contained Vitamin D erroneously, and program managers wanted to document Vitamin D deficiency through a national micronutrient survey conducted in 2010, so that deficiency rates could serve as a national baseline. A gap also occurred the previous February as the premix ordered was not delivered in time [18]. Averages present in Table 4 in columns D and E are calculated in two ways: (1) at the “monthly mill level” to reflect the performance of mills when they possessed premix; and (2) at the “cumulative mill level” to reflect the performance of each mill over the 16-month period assuming the ability to fortify for all months that a mill was operational. Calculating the mill performance averages at the national level was not done as part of the fortification monitoring system; calculations are only included here to illustrate aggregate performance for the 16-month period examined.

Of the 13 mills currently operational in Jordan, 11 mills were equipped with micronutrient premix feeders when this monitoring system began. Two mills, Mill #12 and Mill #13, were not fortifying in 2009 and early 2010. Though Mill #12 was built in 2008, and began milling in late 2009, it did not install a micronutrient premix feeder until mid 2010. Mill #13 on the other hand was under construction during most of 2009, and was able to secure all equipment to begin fortifying in 2010. Of the 11 mills fortifying in April 2010, the monitoring system documented that when premix was available, the mills used an average of 79% of the target premix amount (250 g/MT) and had an average iron concentration of 34 ppm. Though not perfect, these results show that mills in Jordan are able to fortify close to national standards. When examining the national performance during the 16-month period, the months where the premix was not available are included in the average and set to zero. Using this approach, mills averaged 52% of the target premix amount and had an average iron concentration of 21 ppm.

**Table 4:** Monthly mill monitoring results from January 2009 to April 2010.

(A)	(B)	(C)																(D)	(E)
Mill Name	Indicator	Jan-13	Feb-9	Mar-9	Apr-9	May-9	Jun-9	Jul-9	Aug-9 ^a^	Sep-9 ^a^	Oct-9 ^a^	Nov-9	Dec-9	Jan-10	Feb-10	Mar-10	Apr-10	Monthly Mill Average ^b^	Cumulative Mill Average ^c^
**Mill 1**	*Mowahad* flour production (MT)	1336	2413	2990	260	2402	2402	2518	2395	1817	6194	2393	2275	1959	1962	3206	2056	2411	2411
	Addition rate, % target 250 g	97%	PSO	43%	100%	PSO	100%	NRC	PSO	PSO	PSO	88%	97%	107%	92%	84%	92%	90%	60%
	Iron level, PPM	38		36	45		47					36	39	39	60	49	128	52	32
**Mill 2**	*Mowahad* flour production (MT)	5988	5102	5756	6486	5933	5647	5705	4631	3810	5319	5397	5009	5725	5116	5792	4765	5386	5386
	Addition rate, % target 250 g	95%	PSO	38%	94%	84%	78%	NRC	PSO	PSO	PSO	96%	98%	66%	59%	52%	38%	73%	53%
	Iron level, PPM	38		35	38	29	30					39	40	15	32	31	missing	33	22
**Mill 3**	*Mowahad* flour production (MT)	2170	1995	2150	2188	1935	1935	2140	2310	2185	2205	2240	2100	2100	1970	2065	1980	2104	2104
	Addition rate, % target 250 g	83%	PSO	33%	91%	PSO	78%	84%	59%	PSO	PSO	58%	85%	110%	91%	82%	81%	78%	58%
	Iron level, PPM	29		39	35		29	9	20			26	30	45	45	42	40	32	24
**Mill 4**	*Mowahad* flour production (MT)	3861	3802	4211	2777	4536	4536	4381	4415	3536	4915	5021	4832	5000	3000	5000	4500	4270	4270
	Addition rate, % target 250 g	96%	PSO	38%	94%	PSO	88%	NRC	PSO	PSO	PSO	98%	87%	92%	90%	28%	100%	81%	54%
	Iron level, PPM	35		40	39		35					39	34	23	41	40	43	37	23
**Mill 5**	*Mowahad* flour production (MT)	7150	6333	6898	5404	5167	5168	5809	5375	4097	4907	5532	4481	5211	4694	4682	5210	5382	5382
	Addition rate, % target 250 g	96%	PSO	30%	98%	48%	48%	72%	93%	PSO	PSO	81%	85%	77%	87%	64%	69%	73%	59%
	Iron level, PPM	39		34	42	missing	32	missing	missing			34	31	32	36	11	15	31	24
**Mill 6**	*Mowahad* flour production (MT)	2297	2181	2508	2349	2139	2139	2265	2340	2312	2995	3163	3072	2792	2576	2836	2757	2545	2545
	Addition rate, % target 250 g	91%	PSO	32%	85%	PSO	79%	NRC	PSO	PSO	PSO	76%	72%	46%	39%	35%	62%	62%	41%
	Iron level, PPM	30		28	28		27					29	27	14	13	missing	16	24	14
**Mill 7**	*Mowahad* flour production (MT)	6699	6470	6801	6606	6656	6656	6095	4618	3542	3991	4253	3742	4492	3496	4214	3972	5144	5144
	Addition rate, % target 250 g	90%	PSO	41%	95%	PSO	77%	NRC	PSO	PSO	PSO	85%	91%	98%	92%	95%	100%	86%	58%
	Iron level, PPM	30		32	40		34					35	37	19	22	62	25	34	21
**Mill 8**	*Mowahad* flour production (MT)	6602	6044	6474	6323	6595	6595	6320	6049	5289	6561	6873	6536	6485	5982	6604	6348	6355	6355
	Addition rate, % target 250 g	97%	PSO	43%	98%	88%	84%	NRC	PSO	PSO	PSO	97%	99%	86%	47%	45%	50%	76%	56%
	Iron level, PPM	40		39	40	missing	42					40	42	22	21	12	14	31	
**Mill 9**	*Mowahad* flour production (MT)	6674	6018	6727	6563	6631	6631	6538	5989	4751	6194	6447	6175	5733	5734	6380	5960	6197	6197
	Addition rate, % target 250 g	100%	PSO	37%	91%	60%	80%	72%	PSO	PSO	PSO	93%	86%	91%	78%	63%	67%	77%	57%
	Iron level, PPM	42		38	37	18	36	72				42	33	63	25	19	34	38	29
**Mill 10**	*Mowahad* flour production (MT)	20	2295	2233	1989	2285	2285	1906	2481	2270	3058	3312	3167	3291	missing	missing	2818	2386	2386
	Addition rate, % target 250 g	74%	PSO	40%	80%	PSO	83%	NRC	PSO	PSO	PSO	81%	73%	97%	NRC	NRC	103%	79%	49%
	Iron level, PPM	26		37	26		31					37	29	45	28	28	27	31	20
**Mill 11**	*Mowahad* flour production (MT)	758	476	543	573	653	652	708	650	619	756	579	772	1200	800	1200	931	742	742
	Addition rate, % target 250 g	99%	PSO	37%	100%	83%	92%	100%	PSO	PSO	PSO	86%	90%	100%	100%	100%	129%	93%	70%
	Iron level, PPM	35		33	44	40	40	missing				36	41	missing	28	32	46	37	27
**Mill 12**	*Mowahad* flour production (MT)	0	0	0	0	0	0	0	0	0	0	1026	1153	1000	571	1102	1386	390	390
	Addition rate, % target 250 g	NFM	NFM	NFM	NFM	NFM	NFM	NFM	NFM	NFM	NFM	NFM	NFM	NFM	NFM	NFM	NFM	-	0%
	Iron level, PPM	0	0	0	0	0	0	0	0	0	0	0	0	0	0	0	0	-	0
**Mill 13**	*Mowahad* flour production (MT)													2970	2544	2780	2820	2779	2779
	Addition rate, % target 250 g	MUC	MUC	MUC	MUC	MUC	MUC	MUC	MUC	MUC	MUC	MUC	MUC	64%	79%	86%	57%	71%	71%
	Iron level, PPM	MUC	MUC	MUC	MUC	MUC	MUC	MUC	MUC	MUC	MUC	MUC	MUC	missing	25	25	27	25	25
																		TOTAL ^b^	TOTAL ^c^
**Total**	*Mowahad* flour production (MT)	3630	3594	3941	3460	3744	3720	3699	3438	-	-	3853	3610	3689	3204	3822	3500	3605	3605
**Average** ^b^	Addition rate, % target 250 g	93%	-	38%	93%	73%	81%	82%	76%	-	-	85%	87%	86%	78%	67%	79%	79%	
**Average** ^c^	Addition rate, % target 250 g	85%	0%	34%	86%	30%	74%	66%	13%	0%	0%	78%	80%	80%	71%	61%	73%		52%
**Average** ^b^	Iron level, PPM	32	-	33	35	22	32	27	10	-	-	33	32	29	29	29	35	30	
**Average** ^c^	Iron level, PPM	32	0	33	35	9	32	8	2	0	0	33	32	29	29	29	35		21

NOTE: Source of data from mill monitoring reports except underlined figures, which are from the Ministry of Trade; MT = metric tons; g/MT = g of premix per metric ton; PPM = parts per million; PSO = premix stock out; NRC = no report collected; NFM = no feeder in mill; MUC = mill under construction. ^a^ Premix stock outs between August and October 2009 were due to premature addition of vitamin D to the premix; premix with added vitamin D was withheld from the mills at this time in order to provide an opportunity to conduct a baseline study on vitamin D levels before vitamin D was added to premix; ^b^ Average calculated for addition rate (%) and iron level (ppm) only using months for which data are available (*i.e.*, PSO, NRC, MUC, and NFM considered as missing for calculations); ^c^ Average calculated for addition rate (%) and iron level (ppm) using all months (*i.e.*, PSO and NFM are considered equal to 0 for addition rate variable. NRC and MUC are considered missing for the calculations).

There is not always consistency between the addition rate of premix (addition rate as % of target 250 ppm) and iron concentration in the flour samples tested (iron level PPM). For example, there are cases in which the % of target addition rate is low (<80%) and the PPM detected in the flour samples is higher than national standards (*i.e.*, >32.25 PPM iron).

## 6. Discussion

The data presented above demonstrates how the collection of basic data on a regular basis can be compiled to give both a mill-specific and aggregate picture of a fortification program’s performance. Furthermore, it demonstrates how data from such a system can be easily interpreted and acted upon by program managers. For program managers, this regular compilation of the data enables a timely identification of mills not in compliance with fortification standards, allowing for problems to be corrected as they occur. Moreover, it provides useful feedback for decision-making and action processes, including assessing millers’ needs (e.g., for micronutrient premix or spare parts). In the case of Jordan, the system both helped to identify mills that encountered technical issues with the operation of the micronutrient premix feeds and non-compliant mills. When mills experienced technical difficulties, members of the Technical Committee assisted mills to resolve the issues. In cases of non-compliance, the MoH issued government warnings detailing mill owner’s legal obligations to fortify *Mowahad* wheat flour according to government standards. Though this external monitoring system aimed to show monthly results in anticipation of a national micronutrient survey conducted in 2010, difficulties in the procurement/distribution of micronutrient premix meant that gaps in fortification occurred.

The results highlight that a key challenge in implementing the external monitoring system is the person-power required to conduct the various tasks. While routine compilation of flour production and premix utilization data does not require substantial resources, the on-site inspection and collection of flour samples is labor intensive and costly (due to time requirements and petrol costs for visiting mills far from Amman). As all agencies involved in the Technical Committee have competing responsibilities and do not have the human or financial resources to sustain monthly collection of flour samples, less frequent collection of flour samples may be required. As such, quality control visits can be reduced from monthly to bi-monthly or quarterly. Regarding the inconsistency between the addition rate as % of target 250 ppm and iron concentration in the flour samples, the analysis of composite flour samples should, to some extent, mitigate the variability of the iron concentration observed in the final product. That said, the variability in the premix feed rate during the day can affect the iron concentration observed in the final product, particularly if composite flour samples are not made using individual samples collected over an 8-h period [9]. Thus, at times the iron level observed from quantitative testing may be less reflective of the adherence to fortification standards of each mill than premix utilization rates. While removing iron concentration as an indicator from the monitoring system could be considered, it is worth noting that quantitative testing (conformity assessments) provides both a confirmation of the addition rate indicator and can be used to interpret and document the causes of poor compliance. To illustrate: a mill’s poor utilization of micronutrient premix could be caused by consistently low amounts of premix being added (likely due to complications with the micronutrient feeder) or gaps in the use of micronutrient premix (due to the failure to replenish the stock inside feeder). Lastly, the dual indicators also permit program managers to “flag” a mill for further inquiry if the measures are inconsistent.

Jordan provides a straight-forward case study of an external monitoring system used to assess the performance of a relatively small number of wheat flour mills (13 as of 2012) within a small geographic area. For countries where the number of mills is much greater and mills are geographically dispersed, however, an automated system may be favorable.

## 7. Conclusions

Internal (*i.e.*, mill-level) QA processes and QC tests related to fortification can be complemented and verified by external monitoring implemented by government health or food safety agencies. External monitoring of a small number of key indicators can provide program managers with information that is simple to collect, timely, and representative. With information provided by such an external monitoring system, decisions can be based on timely and substantiated evidence.

Although there have been many reports in the peer-reviewed literature describing how to conduct household monitoring and/or impact evaluations of flour fortification programs, there is sparse literature available on how to construct an external monitoring system based on data from factory inspections and technical auditing. As the external monitoring results of a fortification program can greatly inform the design of a household monitoring survey or impact evaluation, improving the quality of and access to external monitoring data can assist evaluators of food fortification programs.

Jordan’s external monitoring system successfully documented the performance of each mill and the entire flour fortification program. In addition, it shows that a simple monitoring system can be implemented and maintained by a relatively short-staffed Nutrition Division and Technical Committee and that the information captured can be easily analyzed, interpreted, and utilized in making programmatic decisions.
